# Modeling the Effects of Urban Design on Emergency Medical Response Calls during Extreme Heat Events in Toronto, Canada

**DOI:** 10.3390/ijerph14070778

**Published:** 2017-07-14

**Authors:** Drew A. Graham, Jennifer K. Vanos, Natasha A. Kenny, Robert D. Brown

**Affiliations:** 1 School of Environmental Design and Rural Development, University of Guelph, Guelph, ON N1G 2W1, Canada; drew.a.graham@gmail.com; 2 Scripps Institution of Oceanography, University of California San Diego, 9500 Gilman Dr, La Jolla, CA 92093, USA; jkvanos@ucsd.edu; 3 Taylor Institute for Teaching and Learning, University of Calgary, Calgary, AB T2N 1N4, Canada; nakenny@ucalgary.ca; 4 Department of Landscape Architecture and Urban Planning, College of Architecture 3137, Texas A&M University, College Station, TX 77843-3137, USA

**Keywords:** landscape architecture, urban design, energy budget modeling

## Abstract

Urban residents are at risk of health-related illness during extreme heat events but the dangers are not equal in all parts of a city. Previous studies have found a relationship between physical characteristics of neighborhoods and the number of emergency medical response (EMR) calls. We used a human energy budget model to test the effects of landscape modifications that are designed to cool the environment on the expected number of EMR calls in two neighborhoods in Toronto, Canada during extreme heat events. The cooling design strategies reduced the energy overload on people by approximately 20–30 W m^−2^, resulting in an estimated 40–50% reduction in heat-related ambulance calls. These findings advance current understanding of the relationship between the urban landscape and human health and suggest straightforward design strategies to positively influence urban heat-health.

## 1. Introduction

The global climate is changing and many areas are experiencing hotter summer weather. More than half the world population is now living in cities and this proportion is expected to continue to increase. Large cities are well known to produce an Urban Heat Island (UHI) where the city is warmer and drier than the surrounding countryside [1,2,3,4,5], and research suggests that vulnerability to heat-related health effects is often greatest within the urban core [5,6]. The combination of global and urban climate change puts human health at risk, particularly during heat waves, or Extreme Heat Events (EHE). A rise in intensity, frequency, and duration of EHEs has been forecast for North America and Europe by the end of the 21st century [7]. Urban residents experience elevated rates of heat-related morbidity and mortality compared with those in rural environments [8,9,10]. Extreme heat is responsible for the highest number of weather-related deaths [8,11]. Two notable and extreme examples include over 400 heat-related deaths in Chicago, IL from a 7-day EHE in July 1995 [12] and nearly 39,000 excess deaths across 12 European countries during a 14-day EHE in August 2003 [13]. Focusing efforts on mitigation of heat-related morbidity is appropriate, considering that it is a preventable condition and a prerequisite for heat-related mortality [14].

Despite the fact that most people know when an EHE is occurring [8,15], their behaviour can be contradictory. They may: (1) not consider themselves to be vulnerable to heat [15]; (2) experience a diminishing perceived threat with increasing EHE duration [8]; (3) lack knowledge of appropriate preventative actions (e.g., hydration, running air conditioning) [8,15]; or (4) avoid preventative actions due to economic factors [8,16]. Improving urban heat-healthiness through identification, detailed landscape analysis, and re-design of high-risk areas could reduce citizens’ exposure to extreme heat in a way that requires less conscious effort on the part of the individual and thus makes strides toward mitigating heat-related morbidity and mortality.

Although mapping vulnerability at a neighborhood or landscape-scale is inherently complex [3], human thermal comfort models, which incorporate a number of biophysical and microclimatic parameters to predict the energy balance of individuals in outdoor spaces, can provide a quantifiable metric for mapping heat stress vulnerability in urban areas [17,18]. Importantly, human thermal comfort models can also be applied to predict how landscape modifications can reduce vulnerability to heat stress [18].

The design of the urban landscape can influence outdoor thermal comfort by modifying the microclimate [17,18,19,20,21]. A study in Phoenix [17] reported that human energy budgets modelled during an EHE correlated negatively with both the amount of open space and vegetation abundance in specific neighbourhoods. In another study, city-wide human energy budgets were strongly correlated to the frequency of heat-related EMR calls during a 3-day EHE in July 2010, in Toronto [22].

This study builds on previous research [23] that reported higher heat-related Emergency Medical Response (EMR) calls related to two physical characteristics of neighborhoods: amount of tree canopy cover and amount of impervious surface. Areas with fewer trees and higher percentages of impervious surfaces received up to five times as many heat-related EMR calls during EHEs. The goal of this study was to further explore the relationship between physical characteristics of neighborhoods and human heat-health through the application of a validated human thermal comfort model at the Census Tract (CT) level during EHEs in Toronto Canada. Landscape architectural design solutions that have been suggested as ways to cool overheated landscapes were tested through modeling and reported in terms of the reduction in expected heat-related EMR calls.

## 2. Materials and Methods

Predetermined criteria were used to select four EHE periods from a historical pool of days classified as either “heat alert” or “extreme heat alert” by the City of Toronto Public Health Department’s Spatial Synoptic Classification system, as previously described in detail [23]. The dates used for analyses were: 27–30 June 2005; 29 July–2 August 2006; 24–27 May 2010; and 29 August–2 September 2010. The seven days preceding (Pre) and following (Post) each EHE were also examined as a baseline for comparison. Five Pre/Post days were classified as “heat alert” and were removed from all analyses. Hourly weather data recorded at Toronto International (Pearson) Airport and Toronto (Buttonville) Airport were assessed for all dates studied [23]. EMR calls for all study dates were analyzed to identify heat-related illnesses by CT. Heat-related EMR calls were defined as a subset of conditions typically related to heat illness, including: breathing problems, cardiac or respiratory arrest/death, non-traumatic chest pain, headache, heat/cold exposure, stroke/cerebrovascular accident, and unconscious/fainting. The rationale for using this subset of reported conditions has been reported previously [23].

The Toronto land cover raster dataset was used to identify potential microclimate-modifying characteristics of CTs. This system used satellite imagery of city surfaces to classify each pixel (resolution = 0.6 m × 0.6 m) into one of eight land classes: Tree Canopy, Grass/Shrub, Bare Earth, Water, Building, Road, Other Paved Surfaces, or Agriculture. Land cover data were extracted for each CT and the resulting attribute table exported as a text file.

To test the accuracy and precision of the extraction method to cover an entire CT, by-CT pixel counts for each land class were converted to areas (in ha). By-CT sums of land class areas were compared to the CT areas generated by the geodatabase (based on the geo-located CT boundary polygons). Differences ranged from 0.00% to 2.77% of CT area, with a mean ± SD of 0.06 to 0.24%. This analysis confirmed reasonable precision (narrow range, low SD) and accuracy (mean close to 0) and thus a reliable and valid method of land cover-by-CT data extraction.

The COMfort FormulA (COMFA) [24,25,26,27,28,29] is a validated model that estimates a human’s energy flux in outdoor environments and reports the balance as an Energy Budget (EB, in W m^−2^). It calculates this through a summation of heat production (from metabolic processes and absorbed short- and long-wave radiation) and loss (through sensible heat loss, emitted long-wave radiation, and evaporative heat loss). There are a variety of human physiological variables, meteorological variables, and physical properties used as inputs for COMFA [24]. The resulting EB provides an estimate of the thermal comfort level experienced by a person in a particular situation.

An interactive Excel version of the COMFA model was used in the study. The output of the model is an EB value, which is an estimate of the overall energy flux of a human (in W m^−2^), calculated as:

(1) EB=M+R−H−L−E

where:

M is metabolic heat conducted to the body surface (W m^−2^),
R is short- and long-wave radiation absorbed by the human (W m^−2^),
H is sensible heat flux (W m^−2^),
L is long-wave radiation emitted by the human (W m^−2^), and
E is evaporative heat loss (W m^−2^).

The hourly weather data input into COMFA included: air temperature (T_a_), relative humidity (RH), wind speed at 10 m (υ_2_), and weather observation. υ_2_ was converted from km h^−1^ to m s^−1^ and converted to a wind speed at a height of 1.5 m (υ_1_, in m s^−1^) by Equation [19]:

(2) $$\upsilon_{1}=\left(\upsilon_{2}\right)\left[\frac{\ln\left(\frac{1.5}{\left(0.13\right)\left(0.8\right)}\right)}{6.65}\right]$$

Hourly weather observations were reclassified into “sunny”, “partly cloudy”, or “cloudy” conditions (coefficients of transmittance = 0.75, 0.40, and 0.25, respectively. Hourly EB values were calculated for the period of 11h00–18h00 daylight savings time (DST) on each study date. This timeframe captures the hottest 8 hours of the day and the time of day when most people are outside (e.g., travelling to and from work), therefore providing an index of the high daytime thermal loads experienced during EHE [22]. It is also the time of day that urban design can have the largest effect on the EB of residents.

The baseline energy budget (bEB) was calculated and used as the standard for comparison. It was represented by the EB estimated under the prevailing conditions and in the measurement environment of the weather stations (i.e., an unshaded, mown turf grass field). Thus, weather parameters for bEB modelling included: no tree presence, 100% sky view factor (SVF), and grass ground cover (albedo of the ground (α_grd_) = 25%). During the EHEs studied, it was estimated that the typical person would try to restrict intensity of physical activity and to wear clothing conducive to staying cool. Therefore, a metabolic activity (M_a_) of 192 W m^−2^ (value for slow walking at a mean velocity of 2.4 km h^−1^) and a clothing ensemble of T-shirt, shorts, socks, and running shoes (static clothing resistance = 50 s m^−1^), respectively, were used for all modelling.

Two Toronto CTs were selected for study based on the following criteria: (1) experienced a high number of EMR calls during EHEs; (2) be in different parts of the city; and (3) be CTs where it is possible that area residents can spend time outdoors (i.e., excluding CTs containing large areas of restricted access, such as airports).

CTs selected for re-design were examined in detail using aerial (ArcMap) and ground-level imagery (Google Earth v. 6.1.0.5001, Google, Mountain View, CA, USA). A scaled base plan of existing conditions was generated for each CT. Climate normals for T_a_, solar radiation, and wind directions for Toronto during the May–September study period [30,31] were examined. Published design guidelines [8,19,20] for cooling of hot weather microclimatic conditions were applied to the design of the selected CTs. The specific cooling interventions prioritized in the proposed master plan designs of the two CTs were:

- Addition of deciduous trees, prioritizing areas to the south and west of high foot traffic routes (e.g., sidewalks, trails) and infrastructure (e.g., buildings <10 stories, parking lots, streets).
- Re-alignment of rows of parking stalls from north–south to east–west to maximize shading of the parking lot.
- Replacement of existing dark coloured (low albedo) asphalt pavements in roadways and parking lots with light coloured (high albedo) concrete pavements.
- Replacement of traditional (dark coloured) roofing materials with either a green roof or light coloured (high albedo) roofing material. Note: although buildings are not accounted for in the calculation of EB in the present study, this design strategy would reduce the building’s emitted terrestrial radiation and its demand for air conditioning, which emits terrestrial radiation when in operation.

Since the goal of the re-design was to offer cooling solutions during EHEs, priority was placed on reducing radiation exposure (short-wave solar and long-wave terrestrial radiation) [20]. Maximizing exposure to the predominant winds during sunny summertime conditions was considered a secondary priority [20], and research suggests that wind alterations have been shown to have minimal cooling effects on an individual’s energy balance during heat waves [18]. During heat waves in Toronto, the humidity is typically very high so very little evaporative cooling could be expected to occur. Air temperature and air humidity were not addressed as they are spatially conservative at the site scale and cannot be modified substantially through urban design [20]. With these strategies in mind, master plans of proposed “cooling” design solutions were generated for each study CT.”

A scaled plan view image (tagged image file format, 300 dpi, greyscale) was generated of each CT in which each land class of interest (Tree Canopy, Grass/Shrub + Agriculture, Bare Earth, Roads, Other Paved Surfaces) was rendered a unique shade of grey. Absolute pixel count of each shade of grey (i.e., land class) was determined by ArcMap and proportional areas calculated. Proportional areas for each CT before (i.e., existing) and after design modifications (i.e., proposed) were used to calculate hourly predicted energy budgets (pEBs) from 11h00 to 18h00 on each EHE day.

Pearson product-moment correlation procedures were used to examine the strength and direction of associations between pairs of weather variables. The normality assumption was assessed using statistical (Shapiro–Wilk’s test) and graphical (stem-and-leaf plot, frequency histogram, Q–Q plot, detrended Q–Q plot, box plot) interpretation methods. The CT-level EMR call data failed normality testing. Spearman rank order correlations (a non-parametric test) were thus run for all correlation analyses involving CT-level call data. Additionally, log transformation resolved normality in these CT-level call data. In some cases, Spearman correlations of non-transformed call data (vs. pEB) were verified against Pearson correlations of log-transformed call data (vs. pEB), achieving very similar results. Assumptions of linearity, lack of outliers, and homoscedasticity were assessed graphically (scatter plots) and were reasonably met in all cases. A significance level of *p* < 0.05 was used. Analyses were performed using SPSS Statistics software (v. 19.0.0, IBM Corporation, Armonk, NY, USA).

## 3. Results

**Figure 1:**
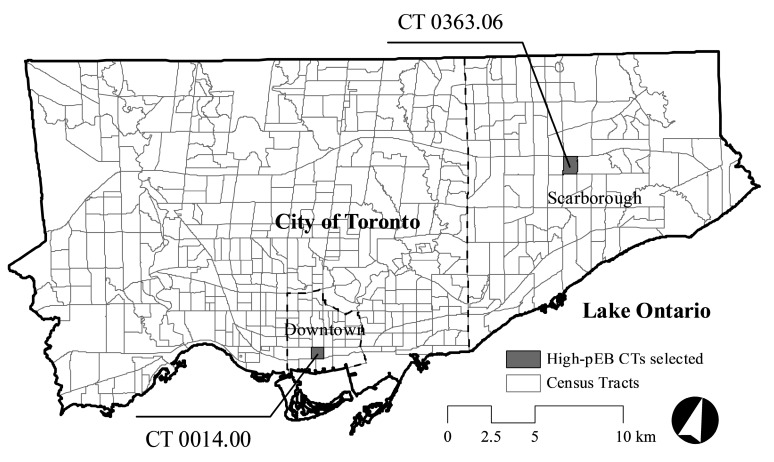
Locations of Census Tracts (CTs) selected for re-design.

Two CTs met the selection criteria: 0014.00 (hereafter referred to as Downtown); and 0363.06 (hereafter referred to as Scarborough). Characteristics and locations of selected CTs are presented in Table 1 and Figure 1, respectively.

Four EHEs occurred in Toronto during the period 2005 to 2010 [23]. The days before (Pre) and after (Post) the EHE were included in the study to provide a comparison. The numbers of each day type (Pre, EHE, and Post) in each of the four EHEs are displayed in Table 2.

Air temperatures (T_a_, T_min_, and T_max_) from 11h00 to 18h00 were significantly higher during EHE compared to Pre and Post within the mean Pearson + Buttonville data (Table 3).

Daily data from all days (i.e., Pre, EHE, and Post, *n* = 16–19) from each of the 4 years were run separately and resulted in many significant correlations and with high strength (i.e., *r* > 0.5) [32] in their relationships (Table 4) that align with previous reports [22,33]. Event-to-event fluctuations exist in both the relationship strength between correlated variables and the presence of statistical significance for the correlations.

Typical weather (i.e., climate normals) during months encompassing the study dates (May–September) exhibits a characteristic peak in T_a_ (Table 5) and amount of bright sunshine (Table 6) during the month of July.

**Figure 3:**
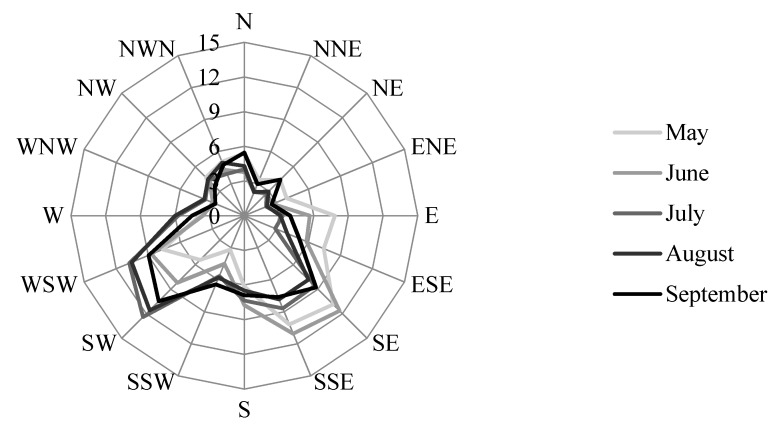
Typical summertime wind directions during sunny conditions at Lester B. Pearson Int’l A weather station for the years 1955–1970. Data are in percentage of time during summer months. Source: {Environment Canada—Atmospheric Environment Services, 1970} [31].

Wind patterns in sunny conditions are of particular interest for designing cooling microclimates. From May to September, typical wind during sunny conditions most often flows from either the SE–SSE or the SW–WSW directions (Figure 3). The SE–SSE direction is more predominant during May and June and the SW–WSW direction is more predominant from July–September. However, designing for a cool microclimate should consider all warm months. On average, from May–September, sunny condition winds are from the SE–WSW 50% of the time. Leaving this range of directions unobstructed would maximize wind flow during summertime sunny conditions.

**Figure A1:**
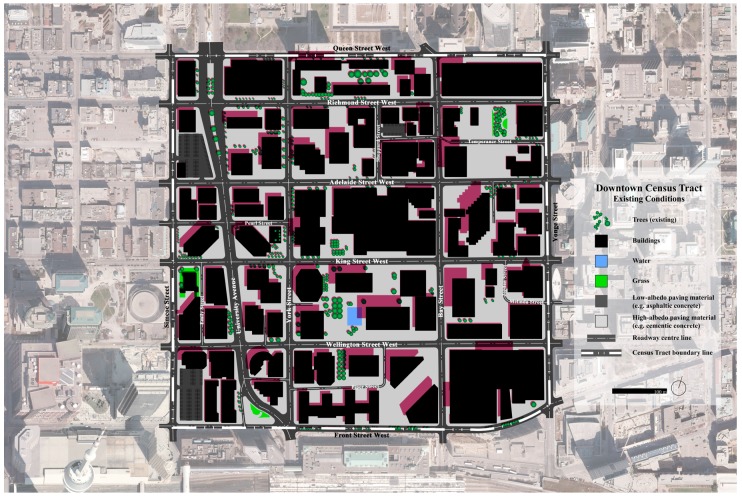
Existing conditions of Downtown.

**Figure A2:**
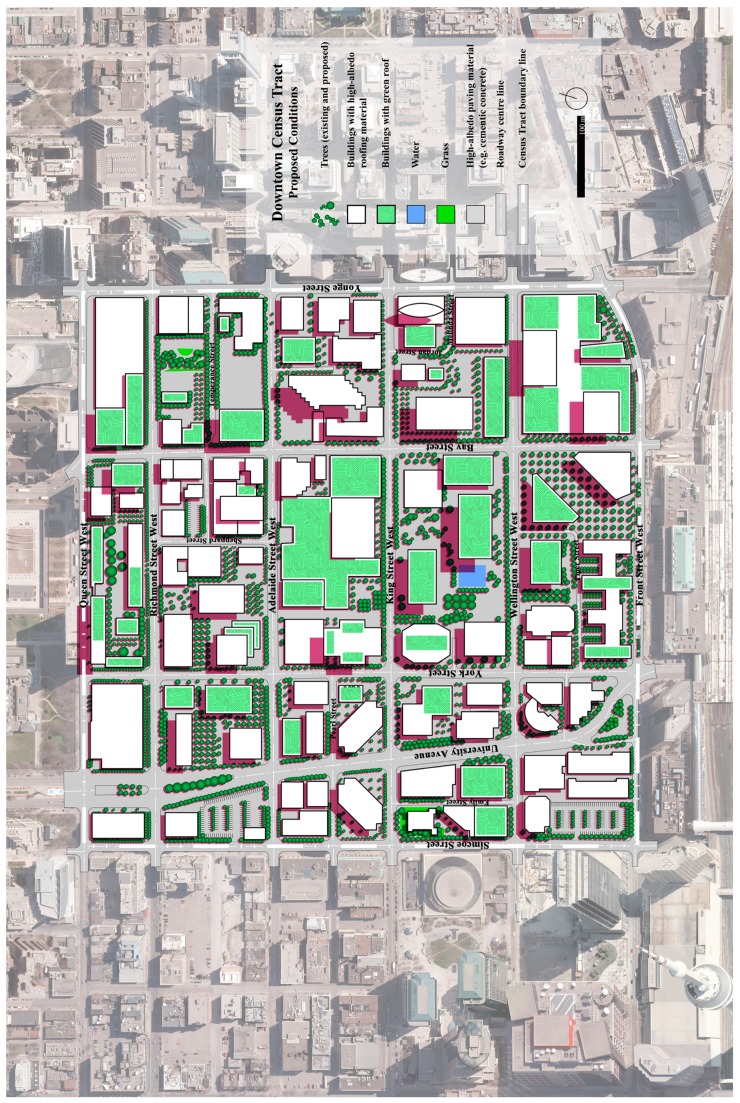
Microclimatic redesign of Downtown.

**Figure A3:**
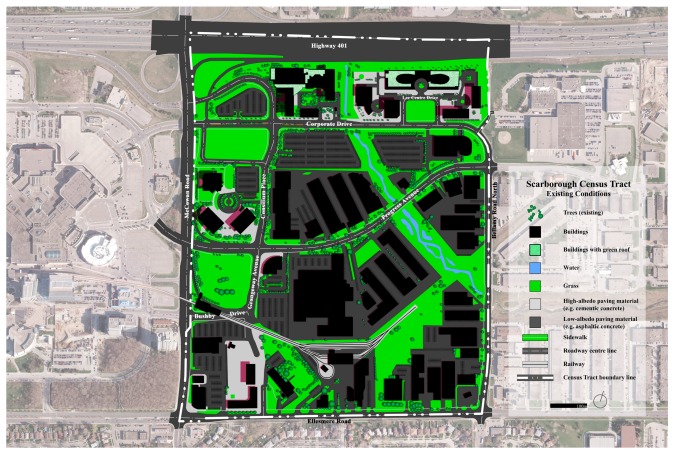
Existing conditions of Scarborough.

**Figure A4:**
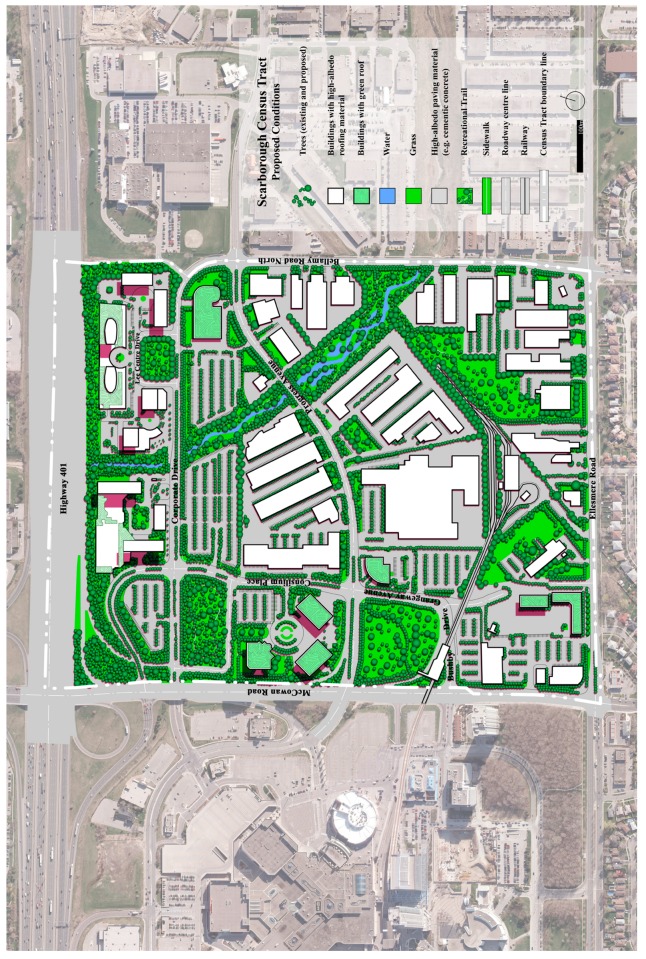
Microclimatic redesign of Scarborough.

The existing microclimatic conditions in Downtown and Scarborough were assessed (Figure A1 and Figure A3) and the landscape “redesigned” through the use of modelling in order to produce more thermally-comfortable conditions during EHE (see example mitigation strategies in [34]). The resulting designs are shown in Figure A2 and Figure A4 and the explanation for the modifications is provided in the following sections.

Ground-level exposure to short-wave solar radiation was reduced in the proposed designs mainly by supplementation of the landscape with deciduous trees (*n* = +2323 for Downtown; *n* = +5266 for Scarborough where n represents the sample size of trees for analysis). While trees allow transmission of the infrared component of solar radiation (~45% of total incoming solar radiation on a clear day), they intercept radiation in the visible spectrum (~45%) [20] to varying degrees depending on species-specific transmissivity [35]. Additional benefits are offered by trees (e.g., habitat, carbon dioxide gas exchange, softening of the urban landscape) compared to solid shade structures that block all direct solar radiation (~90% of total incoming solar radiation on a clear day) [20]. This strategy increased the relative canopy cover in Downtown by 5.3-fold (from 2.2% to 12.0%) and in Scarborough by 6.2-fold (from 3.9% to 24.6%). Specific species were not proposed in the designs, although those with low urban tolerance and with a high ozone-formation potential (e.g., most *Quercus* spp. and *Salix* spp.) should be avoided. Deciduous species were chosen since most of these species defoliate in autumn and would therefore allow solar radiation to be maximized during the winter (which would be desirable).

Placement strategy of proposed trees was to the south and west of areas where humans were most likely to be (e.g., sidewalks, trails) for maximum interception of direct solar radiation during the hottest times of the day. Additionally, proposed trees were placed to the south and west of infrastructure (e.g., buildings, parking lots, streets) in order to minimize the increase in surface temperature (T_sf_) of these elements (and therefore minimize terrestrial radiation emitted by these objects). Trees placed to provide shade to buildings were prioritized for buildings that were <10 stories in height in order to minimize the absorption of solar radiation by the building. Some areas of Downtown that would otherwise have benefitted from shade trees are immediately north of a skyscraper. Since these buildings cast significant shadows to the north, trees were not proposed for these areas. Whenever possible, re-alignment of rows of parking stalls from north–south to east–west (to allow trees to be planted in an east–west orientation, maximizing shading of the parking lot) and addition of planting boulevards in parking lots (both CTs) and along wide streets (Scarborough) were also proposed.

The amount of long-wave terrestrial radiation emitted by most materials is directly related to their temperature [20]. Any strategy that reduces absorption of *short-wave solar* radiation by a material, and consequently its T_sf_, will also contribute to a reduction in *long-wave terrestrial* radiation emitted by the material into the surrounding landscape. Shading from these trees will thus indirectly contribute to reducing a human’s exposure to terrestrial radiation emitted by ground materials that would otherwise heat up more under direct sunshine [25,27,36]. Additionally, the use of lighter-coloured (i.e., higher-α) paving materials has been proposed. These materials reflect more of the incoming short-wave solar radiation and thus decrease the amount of solar radiation absorbed and re-emitted by the ground surface as terrestrial radiation [2,36]. To achieve this effect, the replacement of asphaltic concrete (α_grd_ = 10%, ε = 0.90) with cementic concrete (α_grd_ = 30%, ε = 0.75) is proposed for roadways and parking lots.

Although buildings are not accounted for in the calculation of pEB in the present study, the proposed use of roofing and building materials that better intercept (e.g., green roofs and walls) or reflect (e.g., lighter coloured or reflective materials) incoming solar radiation would help to cool the landscape in two ways. These strategies would reduce both the building’s T_sf_ (and emitted terrestrial radiation) and its demand for air conditioning (which emits terrestrial radiation when in operation). In this light, either green roofs or high-α roofing material was proposed for all buildings that did not have them initially.

Maximizing exposure to predominant winds will work toward maximizing convective and evaporative heat losses by a human [20]. However, this strategy was not prioritized in the proposed designs per se for several reasons:

- convective heat loss is minimized as T_a_ approaches skin temperature (~33 °C) and convective heat *gain* occurs when T_a_ exceeds skin temperature [20],
- mean T_a_ and T_max_ during the hottest part (11h00–18h00) of EHE days in Toronto can reach mid- to high-30s (in °C), respectively, thus minimizing the ability of convective heat loss to cool a person down,
- the predominant winds during sunny summertime conditions are from the SE–WSW in Toronto (Figure 3) and thus inherently conflict with the ideal placement of shade trees to the south and west of outdoor areas populated by humans, and,
- the highly complex, turbulent, and notoriously unpredictable winds in very dense urban landscapes [2,20], such as Downtown, likely do not follow predominant patterns, making it very difficult to strategize for maximizing wind in these areas.
- The compounding effects of high vapor pressure and high temperatures during EHEs in Toronto result in minimal changes to overall energy budgets with higher winds, as the vapor gradient between the above the skin and the air is weak [18].

That said, pruning of low branches will allow ground-level wind to pass under a tree and to reach a human enjoying the cool shade to the north and east of the tree as well as cooling provided through evapotranspiration. An additional layer of complexity occurs in highly dense urban areas (e.g., Downtown) where trees are placed in tight spaces and will likely not reach a typical height at maturity. In summary, in the context of the proposed designs, maximizing wind was a balance between tree maintenance and placement and not a design consideration.

**Figure 4:**
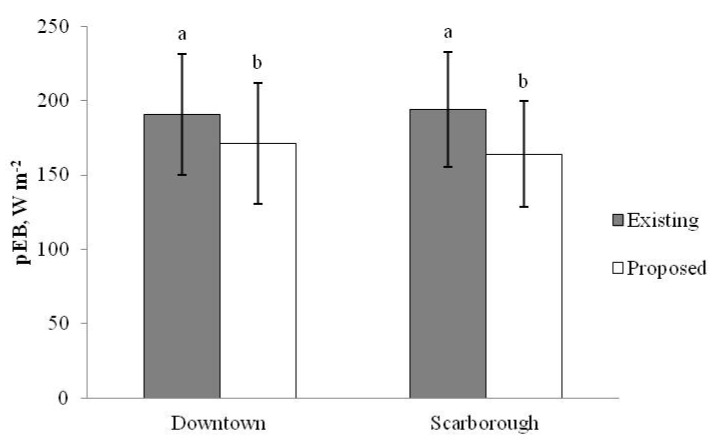
Mean daily (11h00–18h00) pEB on EHE days calculated using the master plans of Downtown and Scarborough before (Existing conditions, grey bars) and after (Proposed conditions, white bars) “cooling” re-design. Data are mean ± SD. Letters “a” and “b” represent comparisons between Existing and Proposed designs within CT, where different letters indicate *p* < 0.0005 for master plans of Existing vs. Proposed conditions (within-CT, by paired *t*-test).

Collectively, the design strategies employed to reduce absorbed solar and terrestrial radiation would work toward lowering a human’s pEB in the urban landscape, especially when the trees are in leaf (i.e., the summer season when EHE typically occur). Comparison of pre- (i.e., Existing conditions in Figure 4) and post-design (i.e., Proposed conditions in Figure 4) pEBs was performed. This involved generation of a raster image of the existing and proposed master plans of each selected CT. These images were analysed for proportional areas of each of the land classes of interest and pEB was calculated. This method was validated by further comparing the pEB calculated for the pre-design (i.e., Existing) conditions of each selected CT to the pEB already calculated for these CTs using the Toronto Land Cover dataset (i.e., land cover data generated from satellite imagery). There was no significant difference between the pEB values using these two methods of assessing pEB of the present-day, existing conditions.

Master plans of proposed design solutions yielded a significantly lower daily pEB on EHE days (as compared to master plans of existing conditions) for both Downtown (−20 ± 2 W m^−2^) and Scarborough (−30 ± 3 W m^−2^). By comparison, these CTs were re-ranked in descending order using the pEB from the proposed master plans. Downtown changed from a ranking of 1st to 176th out of 544 CTs (100th to 62nd percentile) and Scarborough changed from a ranking of 6th to 293rd (88th to 52nd percentile). Findings presented in Graham et al. [23] suggest that these increases in canopy cover would result in reductions in heat-related EMR calls of ~40% in Downtown and ~50% in Scarborough.

## 4. Discussion

The proposed design modifications resulted in meaningful reductions in the daytime pEB during EHEs in the study CTs, nearly achieving a landscape of neutral thermal comfort, and suggest the possibility of post-design reductions in heat-related morbidity in these CTs.

The bEBs, collated across four separate EHEs from 3 different years and across the range of the hot season, are very similar to those reported by Vanos et al. [22] for a 3–5-day EHE in Toronto in July 2005. This is true both for absolute values by period (i.e., Pre, EHE, and Post) and for the ~80 W m^−2^ increase in bEB during the EHE period, as compared to both Pre and Post.

There are other reports relating EB modelling at this spatial scale to various characteristics of the urban landscape [17,18,22]. Harlan et al. [17] reported that modelled EB was positively correlated to population density and negatively correlated to abundance of both vegetation and open space in certain neighbourhoods within Phoenix, AZ. Further, an EB-lowering effect (−26 W m^−2^) of Toronto’s treed urban parks was observed relative to urban streets and open parks during non-EHE conditions [22]. The magnitude of this finding by Vanos et al. [22] is similar to the present finding ~15 W m^−2^ bEB–pEB difference during both Pre and Post periods, especially considering that the latter is an overall average for the entire City of Toronto, and that greater bEB–pEB differences may exist in a given CT or at the sub-CT level. When broadly assessing the impact of urban park characteristics on a person’s thermal comfort (energy budget) across different climate zones using local seasonal climate norms, Brown et al. [18] found that microclimatic landscape alterations in urban parks (e.g., shade trees and structures) that uniformly decreased incoming solar radiation by 50 to 100% could substantially mitigate the personal exposure impacts of heat waves in Toronto, with reductions in bEB during daytime heatwaves ranging from approximately 75 to 130 W m^−2^. These changes are greater than those predicted in the present study due to the more extreme methods applied by Brown et al. [18], which simulated large, uniformly-applied reductions in incoming solar radiation, and focused on the effects during more intense heat waves in the future for long-term planning purposes.

Further, although the modelled changes in surface albedo aided in reducing the energy budgets, related observational studies only controlling for surface albedo show slight increases in the absorbed radiation (or mean radiant temperature) due to a great amount of solar radiation imposed on a human as compared to lower albedo [37,38,39]. The combination of both shade and high-albedo surfaces, however, may cause varying results, yet further observational research in this area is needed.

The methodology and findings presented in this study help to further quantify the impact of interacting UHI mitigation strategies, such as those proposed by Harlan and Ruddell [34] and Stone, Hess and Fumkin [40] within specific CTs. The design solutions offered for Downtown and Scarborough reduced the estimated mean 11h00–18h00 pEB for EHE by 20–30 W m^−2^ (Figure 4). From an absolute EB standpoint, the proposed design modifications brought pEB during EHE in these CTs down from the 190–195 W m^−2^ range (interpreted as *Extreme Caution* and nearly *Danger* according to Harlan et al. [17]) to the 165–170 W m^−2^ range. These changes would result in a much lower risk of heat stress and improved thermal comfort towards the neutral zone of comfort. A person in one of the proposed environments would still be in an EB range that causes them to feel too warm, however they would be much closer to the upper limit of +150 W m^−2^ for the range of EBs categorized as thermally neutral [25,26,28]. Based on the relationship between heat-related EMR calls and EB described herein and elsewhere [28], reducing pEB by 20–30 W m^−2^ on any given day could have a substantial positive effect on human health and well-being in these two CTs. More importantly, the number of proposed trees increases the canopy cover of both CTs up to levels found to be associated with much lower (~40–50%) heat-related EMR call frequencies as compared to existing conditions [23].

## 5. Conclusions

Published recommendations for microclimatically-appropriate design were applied to two CTs in Toronto that had high levels of heat-related ambulance calls during heat waves. The design strategies reduced the energy overload on people by approximately 20–30 W m^−2^, resulting in an estimated 40–50% reduction in heat-related ambulance calls. Post-design pEB was lowered to a level nearing a range of thermal neutrality. This study demonstrated that pEB modelling can aid in the quantification of the thermal comfort benefits of proposed designs and help relate design proposals to beneficial health outcomes. Focusing future efforts on modifying the pEB model to accept the presence of buildings within an acceptable level of error would be a meaningful advancement.

## Figures and Tables

**Figure 2:**
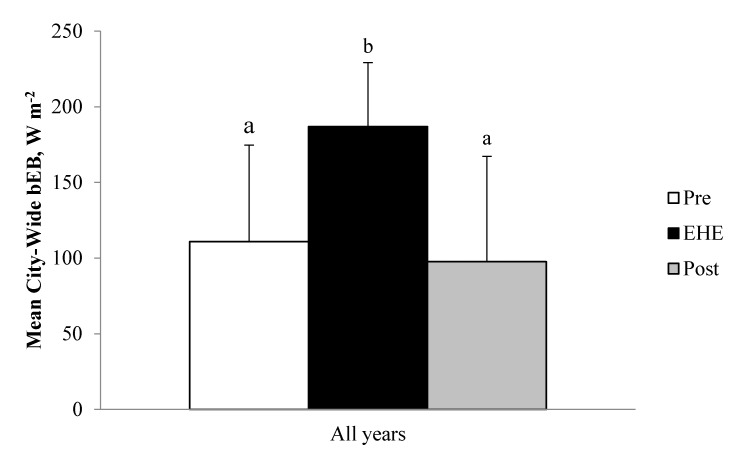
Mean daily city-wide baseline energy budget (bEB) from 11h00 to 18h00 by period (consolidated across all years). Data are mean ± SD. Letters “a” and “b” are between-period comparisons, where different letters indicate statistical significance.

**Table 1:** Characteristics of CTs selected for re-design.

CT Name	Area (ha)	Area of Toronto	Boundaries (N, S, E, W)
Downtown	47.2	Downtown (south central)	Queen St. W, Front St. W, Yonge St., Simcoe St.
Scarborough	82.9	Scarborough (east)	Hwy. 401, Ellesmere Rd., Bellamy Rd. N, McCowan Rd.

**Table 2:** Count of day types by period (Pre, Extreme Heat Events (EHE), and Post) and year.

Day Type	All Years	2005	2006	2010a	2010b
**Pre**	25	6	5	7	7
**EHE**	18	4	5	4	5
**Post**	26	7	7	5	7
**Total**	69	17	17	16	19

**Table 3:** 11h00–18h00 mean weather data from the stations used for modelling (consolidated across all years).

Day Type	T_a_ (°C)	T_min_ (°C)	T_max_ (°C)	RH (%)	υ_1_ (m s^−1^)
Mean Pearson + Buttonville					
**Pre**	23.3 ± 3.8 ^a^	21.0 ± 3.2 ^a^	24.8 ± 4.0 ^a^	56.4 ± 16.0	4.2 ± 1.3
**EHE**	29.7 ± 2.5 ^b^	27.5 ± 2.7 ^b^	31.2 ± 2.6 ^b^	53.8 ± 9.9	4.2 ± 1.5
**Post**	23.3 ± 3.9 ^a^	21.4 ± 3.9 ^a^	24.6 ± 4.0 ^a^	56.0 ± 14.8	5.2 ± 2.2

Data are mean ± SD. ^a,b^ within-station (between-period) comparisons, where different superscript letters (within a measurement) indicate statistical significance. Absence of superscript letters indicates that the main period effect was not statistically significant. T_a_ (air temperature), T_min_, (minimum air temperature), T_max_ (maximum air temperature), RH (relative humidity), υ_1_ (wind speed at 1.5m). City-wide bEB across all years was significantly elevated (+70 to 80 W m^−2^) during EHE from both Pre and Post periods (Figure 2). Pre and Post periods were not significantly different from each other.

**Table 4:** Pearson correlation coefficients (*r*-values) and significance (*p*-values) for city-wide analyses of the bEB–EMR call relationship across all days (Pre, Extreme Heat Events (EHE), and Post, *n* = 16–19) within individual years.

Study Period	City-Wide bEB (8 h)		Mean T_a_ (24 h)		Mean T_a_ (8 h)		T_max_ (24 h)		T_min_ (24 h)	
	*r*	*p*	*r*	*p*	*r*	*p*	*r*	*p*	*r*	*p*
2005										
Total calls	0.432	0.083	**0.733**	**0.001**	**0.692**	**0.002**	**0.683**	**0.003**	**0.637**	**0.006**
Heat-related	0.455	0.066	**0.590**	**0.013**	**0.598**	**0.011**	**0.600**	**0.011**	0.474	0.054
2006										
Total calls	**0.513**	**0.035**	**0.395**	**0.117**	0.381	0.131	0.359	0.157	**0.302**	**0.238**
Heat-related	**0.616**	**0.009**	**0.557**	**0.020**	**0.567**	**0.018**	**0.555**	**0.021**	**0.382**	**0.130**
2010a										
Total calls	0.401	0.124	**0.592**	**0.016**	**0.526**	**0.036**	**0.499**	**0.049**	**0.495**	**0.051**
Heat-related	0.338	0.201	0.368	0.161	0.376	0.151	0.338	0.201	0.172	0.525
2010b										
Total calls	**0.627**	**0.004**	**0.710**	**0.001**	**0.700**	**0.001**	**0.690**	**0.001**	**0.501**	**0.029**
Heat-related	0.289	0.230	0.535	0.018	**0.459**	**0.048**	**0.459**	**0.048**	0.438	0.061

Bolded values indicate statistically significant correlation coefficients (*p* < 0.05).

**Table 5:** Typical summertime air temperatures at Lester B. Pearson International Airport weather station for the years 1971–2000. Source: {Environment Canada, 2012} [30].

Air Temperature	May	June	July	August	September
Mean T_a_, °C	12.9	17.8	20.8	19.9	15.3
T_min_, °C	6.9	11.9	14.8	14.0	9.6
T_max_, °C	18.8	23.7	26.8	25.6	21.0

**Table 6:** Typical summertime bright sunshine at Toronto weather station (+43.6667°, −79.3774°; 112.5 m elevation) for the years 1971–2000. Source: {Environment Canada, 2012} [30].

Summertime Bright Sunshine	May	June	July	August	September
Total bright sunshine, h mo^−1^	229.1	256.2	276.2	241.3	188.0
Frequency of measurable bright sunshine, d mo^−1^	28.1	28.3	30.0	29.6	27.2
Relative amount of bright sunshine, % of daylight hours	50.3	55.5	59.1	55.7	50.0
